# Did the Proband Have Thalassemia Intermedia or Severe Thalassemia Trait?

**DOI:** 10.5505/tjh.2012.91069

**Published:** 2012-03-05

**Authors:** Şinasi Özsoylu

**Affiliations:** 1 Retired Professor of Pediatrics, Hematology, and Hepatology, Ankara, Turkey

## TO THE EDITOR

HBB: (c*+96T>C (3’UTR+1570 T>C) on the mildβ-thalassemia intermedia pheonotype, in the recent issueof the Journal (2011; 28: 219-222). I congratulate theauthors for exploring at the molecular level at least oneof the thalassemia minima that fits well with the presentclinical thalassemia nomenclature. Based on their clinicaldescription, I would not consider the proband as thalassemiaintermedia, as until 20 years of age he did notrequire blood transfusions [1,2]. The mother had thalassemiatrait, the father had thalassemia minima, and theproband clinically had severe thalassemia trait, as he hadmild hepatosplenomegaly and was compound heterozygote(thalassemia trait[+] and carrier of HBB: C*+9,6 T>Cmutation).

I would like to suggest the following arbitrarily modifiednomenclature for clinical thalassemia syndromes:

**Thalassemia major** (according to transfusion requirement):

a) Severe: transfusion interval less than 1 month;

b) Moderate: transfusion interval more than 1 month.

**Thalassemia intermedia** (according to transfusionrequirement):

a) Moderate: transfusion interval less than 1 year;

b) Mild: transfusion interval more than 1 year.

**Thalassemia trait:**

a) Severe: with mild hepatosplenomegaly;

b) Mild: without hepatosplenomegaly;

**Thalassemia minima:** absence of clinical and hematologicalfindings.

**Sincerely**

**Şinasi Özsoylu, MD**

Retired Professor of Pediatrics, Hematology, and HepatologyHonorary Fellow of the American Academy of Pediatrics since1995Honorary Member of the American Pediatric Society since 1993 

## CONFLICT OF INTEREST STATEMENT

The authors of this paper have no conflicts of interest,including specific financial interests, relationships, and/or affiliations relevant to the subject matter or materialsincluded.

## REPLY

First of all we thank Professor Dr. Özsoylu for his contributionand interest in our report. Intermedia is a betathalassemia group characterized by severity varying fromthalassemia major to asymptomatic carriers. It seems thataccording to clinical classification it is possible to describeour patient as severe thalassemia trait rather than mildthalassemia intermedia. On the other hand, the primaryreason we consider our patient as thalassemia intermediais that he is compound heterozygous for 3’UTR+1570 T>Cand Cod 8(-AA) beta-globin gene mutations. We thinkmild thalassemia intermedia is the appropriate descriptionof our patient, considering that he has two differentmutations on his 2 different copies of the beta globingene, as well as mild phenotypic findings. We think thatthe description of severe thalassemia trait is more suitablefor patients with only 1 beta globin gene mutation withclinical findings in addition to the thalassemia minor phenotype.

Again, we appreciate your letter, as it afforded us theopportunity to explain in greater detail our rationale forthe use of genetic data in addition to clinical criteria in theclassification of thalassemias.

## References

[1] Bilgen T, Canatan D, Arıkan Y, Yeşilipek A, Keser İ (2011). The effect of HBB: c.*+96T>C (3’UTR +1570 T>C) on the mild b-thalassemia intermedia phenotype. Turk J Hematol.

[2] Özsoylu Ş (2001). Thalassemia trait as thalassemia intermedia. Am J Hematol.

